# Are Mid-Adolescents Prone to Risky Decisions? The Influence of Task Setting and Individual Differences in Temperament

**DOI:** 10.3389/fpsyg.2019.01497

**Published:** 2019-07-10

**Authors:** Corinna Lorenz, Jutta Kray

**Affiliations:** Department of Psychology, Development of Language, Learning, and Action, Saarland University, Saarbrücken, Germany

**Keywords:** experimental risk-taking, risky decision-making, adolescence, affective task setting, individual differences, temperament, age trends

## Abstract

Recent developmental models assume a higher tendency to take risks in mid-adolescence, while the empirical evidence for this assumption is rather mixed. Most of the studies applied quite different tasks to measure risk-taking behavior and used a narrow age range. The main goal of the present study was to examine risk-taking behavior in four task settings, the Treasure Hunting Task (THT) in a gain and a loss domain, the Balloon Analogue Risk Task (BART), and the STOPLIGHT task. These task settings differ in affective task moderators, like descriptive vs. experienced outcomes, anticipation of gains vs. losses, static vs. dynamic risk presentation, and time pressure vs. no time pressure and were applied in a sample of 187 participants from age 9–18. Beneath age trends, we were interested in their association with individual differences in approach behavior, venturesomeness, impulsivity, and empathy above age, gender, and fluid intelligence. Our findings revealed that risk-taking behavior is only low to moderately correlated between the four task contexts, suggesting that they capture different aspects of risk-taking behavior. Accordingly, a mid-adolescent peak in risk propensity was only found under time pressure in the STOPLIGHT that was associated with higher impulsivity and empathy. In contrast, risky decisions decreased with increasing age in task settings, in which losses were anticipated (THT Loss), and this was associated with higher cognitive abilities. We found no age differences when gains were anticipated, neither in a static (THT Gain) nor in a dynamic task setting (BART). These findings clearly suggest the need to consider affective task moderators, as well as individual differences in temperament and cognitive abilities, in actual models about adolescent development.

## Introduction

Recently, there is an immense increase in studying developmental changes in cognitive, emotional, and social functioning throughout adolescence (for reviews, see Spear, 2000; Steinberg, 2008; Blakemore, 2012; Crone and Dahl, 2012; Shulman et al., 2016). Several research groups have also investigated the association between the development in specific brain regions and decision-making behavior in adolescence (Spear, 2000; Dahl, 2004; Casey et al., 2008; Steinberg, 2008; Smith, Chein, and Steinberg, 2013; Laube and van den Bos, 2016; Luna and Wright, 2016; Sherman et al., 2017). On the basis of such findings, recent theories and models about the neurobiological development in adolescence have proposed divergent developmental pathways over the course of adolescence: an early-maturing incentive-processing system (or socioemotional system) and an only gradually developing cognitive control system may explain why risky and potentially harmful impulses specifically arise in mid-adolescence. Accordingly, while the socioemotional system strengthens motivation to pursue rewards in adolescence, the cognitive control system is not yet matured enough to restrain impulses to achieve rewards and to seek for sensations (cf. Dual Systems Model, Steinberg, 2008; Maturational Imbalance Model, Casey et al., 2008; Driven Dual Systems Model, Luna and Wright, 2016; and Triadic Model, Ernst, 2014). These neuroscientific insights into brain development over the course of adolescence (Giedd et al., 1999; Sowell et al., 2002; Paus, 2005; Casey et al., 2008) might explain the adolescent-specific tendency for exploration and higher risk-taking, as well as the rise in mortality rates during mid-adolescence (see Dick and Ferguson, 2015). Given this, there is a strong need to better understand under which situations higher risk-taking is induced in mid-adolescents, so that in the last decade, a number of quite different laboratory tasks have been created to measure different aspects of risk-taking behavior (for a review, see Defoe et al., 2015).

In such decision-making tasks, adolescents are usually confronted with decisions to engage in gambles for outcomes that differ in their value and probability of occurrence. Risk-taking is, thus, defined as the tendency to choose the option with a higher variability in the range of possible outcomes (cf. Defoe et al., 2015). For instance, preferring gamble options (e.g., 30% vs. 70% chance to win 10€ or nothing) over safe options for which outcomes are known and stable (e.g., a safe win of 2€). Meanwhile, there is also empirical evidence that stands in contrast to the assumptions of neurobiological developmental models, as adolescents did not show the highest tendency for risk-taking (e.g., Spear, 2000; Dahl, 2004; Willoughby et al., 2013). Therefore, a recent meta-analytic review aimed at investigating age differences in several behavioral risk-taking tasks between children (aged 5–10 years), early- and mid-adolescence (aged 11–13 and 14–19 years, respectively), and adults (aged 20–65 years, Defoe et al., 2015). This study indicated that adolescents take more risks than adults do, but only show a higher tendency for risk-taking than children under specific context conditions and characteristics of decision-making tasks.

### The Impact of the Task Setting on Decision-Making in Adolescence

At first, the way risky situations are created is thought to influence decision-making. One type of task setting investigates decision-making behavior for which information about outcomes and their probabilities is given and thereby enables individuals to calculate the profitability of options. These types of tasks are termed description based (e.g., Hertwig et al., 2004; Hertwig and Erev, 2009). Decision-making tasks in which all outcome probabilities are known have usually been seen as less-affective contexts and engage cognitive abilities, like the calculation of expected values (Figner et al., 2009; Defoe et al., 2015). However, associated cognitive abilities are still developing over the course of adolescence (e.g., Luna et al., 2004); thus, children and adolescents may not fully make use of the given descriptive information. Accordingly, two studies compared adolescents to adult tendencies to take risks in a description-based decision-making task, the so-called CUPS task (Levin et al., 2007). While adolescents (aged 14–17 years) and emerging adults (aged 18–21 years) showed no differences in risk-taking propensity (Galván and McGlennen, 2012), middle-aged parents (mean age = 45 years) showed a different choice behavior than their children (aged 8–17 years) in this task (Levin et al., 2014). More specifically, adolescents took more risks than middle-aged parents did. Thereby, parents indeed tended to be more sensitive to expected values than adolescents, and this sensitivity was, in turn, associated with numeracy abilities (Levin et al., 2014). Hence, age, or life experience, and the consequential development of cognitive abilities seem to influence decision-making in description-based tasks.

In addition, the task setting can be influenced by affective task factors, such as the expectation of positive or negative outcomes (e.g., monetary wins or losses, respectively). Especially, adolescents are thought to be biased by the hyperactive socioemotional system to pursue the potentially most rewarding choice (Casey et al., 2008; Steinberg, 2008; Ernst, 2014; Luna and Wright, 2016). However, most findings in the developmental decision-making literature are limited to the effects of different kinds and degrees of gains, while the investigation of the impact of negative outcomes (losses) has been neglected (Kray et al., 2018). This is somewhat surprising, as according to the influential prospect theory (Kahneman and Tversky, 1979), risky decision-making differs depending on whether positive (gains) or negative outcomes (losses) can be expected. For instance, this assumption holds true for adults, as they have been shown to take more risks to prevent losses than to maximize gains in many decision contexts (for a review, see Barberis, 2013). For instance, the CUPS task distinguishes between gain and loss situations. In this task, middle-aged adults took more risks to prevent losses than to win money, while adolescents did not differentiate as much between gain and loss situations as adults did (Levin et al., 2014). Nonetheless, most age groups were rather risk-seeking for potential losses with known outcome probabilities (Reyna et al., 2011; Levin et al., 2014; van den Bos and Hertwig, 2017). One study furthermore showed that the proportion of risk decisions decreased from childhood to early adulthood for losses (aged 8–22 years) but increased for gains in adolescence only (van den Bos and Hertwig, 2017). In sum, the valence of outcomes may influence decision-making in adolescence as well.

Beneath these considerations, potentially risky decisions in everyday life seldom rely on fully known probabilities. Therefore, particularly in the adolescent literature, researchers aimed to raise the ecological validity of experimental decision-making tasks by inducing ambiguity about upcoming positive or negative outcomes and their probabilities. In these so-called experienced-based task settings, the outcome probabilities are unknown and have to be learned through exploration. As such, to learn about outcome probabilities in these tasks, one has to actively engage in risks while only being encouraged to do so by motivators, like gains in money or time. Thereby, experience-based tasks also differ in the way they induce an affective and arousing task setting. In some task settings, the risk levels change dynamically after each decision. For instance, in the Balloon Analogue Risk Task (BART; Lejuez et al., 2002), participants decide to pump balloons when pumping behavior is rewarded, but balloon explosions cause losing all previous gains. Hence, after each decision to inflate the balloon, instead of saving previous gains, the value of outcomes (larger wins) but also the risk for the balloon to explode increase. In other task settings, the risk level remains stable, but the situation becomes arousing through time pressure that is induced for each of the decisions. For instance, one maneuvers a car through multiple intersections for which traffic lights turn yellow when approaching in the STOPLIGHT task. As such, crossing the intersections (GO-decisions) instead of stopping at the lights saves time, but in half of the intersections, the lights turns red beforehand and participants cause accidents in doing so. Thus, participants must choose the most profitable option in a gamble between winning and losing time to earn money (Chein et al., 2011), or reach a social event in a timely fashion (Steinberg et al., 2008).

Decisions that are based on previous experiences are associated with emotion-based learning and should be more affectively arousing (Figner et al., 2009; Defoe et al., 2015). Given that adolescents are thought to be specifically sensitive to affectively engaging situations (e.g., Steinberg, 2008), they may also show more risky decisions in experienced-based than description-based task settings. Indeed, there is some evidence for risk propensity to be highest in adolescents as compared to children and adults in experienced-based task settings. For instance, an inverted U-shaped developmental trend has been found for experienced-based tasks (aged 8–25 years, Braams et al., 2015; aged 10–30 years, Duell et al., 2018), such as the BART and the STOPLIGHT task. Furthermore, adolescents (aged 14–17 years) took more consecutive risk decisions than middle-aged adults (aged 35–55 years) and showed to be specifically sensitive to previous outcomes in the BART (Mitchell et al., 2008). Similarly, early- to mid-adolescents (aged 10–11 years, 12–13 years, and 14–15 years) took more risky decisions than older age groups (aged 16–30 years) in the STOPLIGHT task (Steinberg et al., 2008). Moreover, both task settings showed adolescent-specific influences on risky decisions, such as an association with real-life risk-taking behavior, like dangerous driving under peer presence (Chein et al., 2011), and other health risk behavior (Lejuez et al., 2003; Kim-Spoon et al., 2016). However, little is known about the specific influence of dynamic risk levels and induced time pressure on adolescent decision-making, though adolescents are thought to be more aroused by and take more risky decisions under contextual motivators (e.g., peer observation, Chein et al., 2011) and stressors (for a review, see Galván and Rahdar, 2013). In a recent study, Duell et al. (2018) investigated age trends of the STOPLIGHT task (static risk level with time pressure) and the BART (dynamic risk level without time pressure) in a broad age sample from childhood to mid-adulthood (aged 10–30 years). They found risk-taking in both tasks to develop in an inverted U-shape but showed differing slopes for the two tasks across adolescence. To sum up, it seems that developmental trends in risk-taking throughout adolescence depend on the type of task setting, like whether the expected values for each decision are known or unknown, whether gains or losses can be expected, whether risk itself changes throughout the task, or whether time pressure is induced.

### Relations Between Individual Differences in Temperament and Risk-Taking

A second goal of the present study was to also consider individual differences in temperament that may explain individual differences in risk propensity beyond age. As such, temperament has been proposed to reflect innate characteristics that influence behavior already early in life, while personality rather depicts traits that are acquired in interaction with the environment. However, there is reason to believe that temperament and at least some personality measures share an endogenous nature and, thus, an intrinsic maturation (for a review, see McCrae et al., 2000), and that a clear dissociation between temperament and personality is not reliable. We here refer to temperamental differences instead of the overarching term personality, as we intend to describe basic dispositions that influence adolescent behavior rather independent from life experiences. Thereby, individual differences have been assumed to be associated with real-life risk-taking, while reviews on the role of temperament and personality in experimental decision-making showed inconsistent and contradictory findings (Appelt et al., 2011). Appelt et al. (2011) also pointed to the theoretical and methodological shortcomings of not integrating personality measures in decision-making research and encouraged future studies to consider them on the theoretical basis of various factors. Hence, for the purpose of the present study, we included different temperament factors that have been found to be related to risk-taking behavior in previous studies.

On the one hand, adolescents show a tendency to engage in novel and exciting experiences regardless of potential risks, also known as sensation seeking (Zuckerman, 2007). An associated personality cluster is approach behavior, which is defined as the motivated behavior to pursue potentially rewarding situations. Captured by the Behavioral Activation System (BAS; Gray, 1972), behavioral approach has also been associated with activity in brain regions known for their role in reward processing [nucleus accumbens (Nacc), Urošević et al., 2012; Braams et al., 2015]. Moreover, higher BAS sensitivity has been linked to substance use, dangerous driving, and risky sex in adolescence (e.g., Loxton and Dawe, 2001; Knyazev et al., 2004; Reyna et al., 2011). Despite its rapid rise in adolescence (e.g., Duell et al., 2016), sensation-seeking tendencies do not seem to capture socioemotional imbalance (cf. Romer et al., 2017), as it has been shown to be positively correlated with indicators of executive function (e.g., Zuckerman, 2007).

On the other hand, sensation-seeking tendencies have to be distinguished from the temperamental factor impulsivity that rather reflects decisions for immediate urges without adequately considering potential consequences. Impulsive tendencies are low to moderately associated with sensation seeking, or BAS, as it also peaks during adolescence (Collado et al., 2014; Shulman et al., 2015). However, impulsivity is inversely correlated to executive function, like working memory. Moreover, adolescents with high behavioral approach tendencies might more likely explore risk behaviors, but adolescents with impulsive tendencies are more likely to experience maintained health risk behaviors across development, like addiction (cf., Romer et al., 2017). Nonetheless, many self-report questionnaires capture different facets of “impulsive” behavior. For instance, it can be distinguished between the factor impulsiveness that rather reflects the tendency to act rashly without considering consequences, and the factor venturesomeness that is a characteristic of people who are conscious about potential risks and are willing to take them (I6, Eysenck and Eysenck, 1978). As such, the factor impulsivity might reflect tendencies to engage in risks as one does not consider potential consequences or is less capable in doing so. Though, venturesomeness rather depicts a tendency to engage in situations for which risk is known and is an inherent characteristic (e.g., bungee jumping).

In addition, it has been argued that the most salient types of rewards in adolescence are in the social domain (social feedback like being admired, included, or excluded, or positive and negative emotions; Crone and Dahl, 2012). In support of this view, social contexts like peer presence have been shown to have an age-differential effect, with adolescents taking more risks in these situations than other age groups (for a review see Kray et al., 2018). Correspondingly, adolescence is thought as a period of heightened social–affective engagement and sensitivity (Crone and Dahl, 2012) that might promote empathic responses (Blakemore and Mills, 2014) and, thus, the gradual development of empathic skills over the course of adolescence (e.g., Allemand et al., 2015). Hence, individual empathic functioning might also be predictive of risk-tendency in youth.

### Goals of This Study

In sum, it seems that developmental trends as well as the occurrence of age differences in decision-making tasks vary with the type and characteristic of the task setting. So far, most studies rely on one task setting and a comparison of two or three age groups, which does not allow to draw conclusions about differential influences of task contexts on age differences in the transition from childhood to adulthood (cf. Defoe et al., 2015; Kray et al., 2018).

Therefore, the first goal of this study was to examine whether the type of decision-making task modulated age differences in risk-taking. In order to achieve these goals, we collected data from a relatively broad age sample ranging from 9 to 18 years, which allows to test for linear or quadratic age trends in decision-making. To keep the continuous nature of the age variable and to determine at which age differences between age groups are still significant, we stratified participants into five age groups: 9–10, 11–12, 13–14, 15–16, and 17–18 years. In order to examine whether age differences in risk-taking are modulated by the type of decision-making task, we analyzed age trends in four widely used decision-making contexts: a modified version of the CUPS task [termed in the following Treasure Hunting Task (THT)] in a gain and a loss domain, the BART, and the STOPLIGHT task. We selected these tasks in order to determine whether potentially affective task moderators, like a) description- vs. experience-based outcome probabilities, b) incentive valence in description-based task settings (gains vs. losses), or c) dynamic risk level without time pressure vs. static risk level under time pressure in experience-based task settings, modulate age differences in risk-taking throughout adolescent development.

As a first task, we applied a modified version of the CUPS task, the THT, which reflects so-called description-based decision-making, as decisions are taken under known risk. The THT, moreover, allows us to examine whether age differences in risk-taking are influenced by the valence of anticipated gains and losses. In this task, participants made decisions either in a gain block, in which they could win money (THT Gain), or a loss block, in which they could lose money (THT Loss). On the one hand, we expected risk propensity of the THT to decline over age in the loss domain, as the generally risk-seeking tendencies in such task settings have been found to show a linear decrease over the course of adolescence (e.g., van den Bos and Hertwig, 2017). Regarding the hypothesized reward sensitivity of adolescents and given indices in the literature (for high reward condition; Reyna et al., 2011; van den Bos and Hertwig, 2017), we expected risk propensity in the THT Gain, on the other hand, to show an inverted U shape across adolescence. That is mid-adolescents should take the highest propensity of risk decisions in gambles for gains. In contrast, decisions are taken under ambiguity in experience-based decision-making task, as not all outcome probabilities are known. Therefore, we expected risk propensities to show an adolescent-specific peak in the BART (Braams et al., 2015; Duell et al., 2018) and STOPLIGHT task (Duell et al., 2018). Yet, differences in risk and outcome presentation [dynamic risk level without time pressure (BART) vs. static risk level under time pressure (STOPLIGHT)] might also lead to differential developmental patterns over the course of adolescence for the two experience-based tasks (cf. Duell et al., 2018).

As a second goal of the present study, we determined whether individual differences in temperament, such as approach behavior, impulsivity, venturesomeness, and empathy could explain individual differences in experimental risk-taking beyond age, as these factors have been found to be related to decision-making in a number of studies (see Appelt et al., 2011 for a review). Moreover, we also considered gender and individual differences in fluid intelligence. Male adolescents have consistently been found to engage in higher levels of real-life risk behaviors (e.g., Byrnes et al., 1999; Harris et al., 2006), while higher risk propensity has been shown for male adults in some experimental task contexts, like the BART (Lejuez et al., 2002; Cazzell et al., 2012). However, other task contexts in the adolescent literature revealed no moderator effect of gender (Steinberg et al., 2008; Figner et al., 2009). Given these inconsistent findings, we controlled for possible gender differences in the prediction of risky decision-making across adolescent development, especially as most studies do not provide their results separately for males and females in the literature (cf. Defoe et al., 2015). Furthermore, referring to neurodevelopmental imbalance models, individual differences in cognitive abilities might be associated to tendencies in risky decision-making that reflect an increase in experience and cognitive abilities with age. Usually stated as a factor that decreases risk-taking in these models, findings concerning financial choices assumed that intelligence rather predicts heightened risk-taking behavior, or rather less risk aversion, in the sense of an optimal choice behavior to maximize outcomes (e.g., Donkers et al., 2001; Benjamin and Shapiro, 2005). Moreover, a related factor, namely, numeracy, accounted for differences in sensitivity to expected values, thus in the advantageousness of risk choices between middle-aged adults and adolescents in experimental decision-making (Levin et al., 2014). Therefore, we were interested in the role of individual differences in fluid intelligence in predicting risky decisions across adolescence. In sum, beyond age, we considered gender, as well as individual differences in temperament and fluid intelligence, as predictors for individual susceptibility to risky decisions in experimental decision-making (Appelt et al., 2011; Lauriola et al., 2014; Frey et al., 2017). However, it is an open question whether they differentially explain risky behavior in the four different decision-making settings.

## Materials and Methods

### Participants

Overall, 193 children and adolescents between age 9 and 18 were recruited for this study from a subject pool of our research unit at Saarland University, as well as via flyers and newspaper advertisements. Participants received 8€ per hour as monetary compensation and a small reward that they could choose themselves at the end of one session, measuring cognitive performance and decision-making. Informed consent was given by the participant’s parents or themselves when they were 18 years or older. A local ethic committee at Saarland University gave ethical approval for the project “The Influence of Motivational Processes on Developmental Changes in Adaptive Behavior.”

Five participants were excluded from the analysis of the decision-making tasks because of missing data in one or more tests and tasks. To control for outliers, we first performed tests for uni- and multivariate normality for each of the five age groups: 9–10, 11–12, 13–14, 15–16, and 17–18 years. To this end, we computed Mahalanobis *D*^2;^ probability values for all dependent measures, and if *D*^2^ probability values were lower than 0.001, cases were excluded from the analysis. This was the case for one participant in the 13- to 14-year-olds. Thus, the final sample consisted of 187 participants. Table 1 shows the characteristics of the final sample, including the number of participants in each of the five age groups, gender ratio, socioeconomic status (SES), and two intelligence subtests, one from the fluid domain and one from the crystallized domain of intelligence (for a description of these variables, see the next section). Neither the gender ratio nor the SES differed significantly across the age groups (*p* = 0.12, *p* = 0.67, respectively). In line with results in the literature, we found an increase in reasoning, *F*(4,182) = 22.87, *p* < 0.001, = 0.34, and verbal knowledge, *F*(4, 182) = 36.44, *p* < 0.001, = 0.45, with increasing age (e.g., Li et al., 2004; Nook et al., 2017).

**Table 1:** Descriptive statistics, control, and self-report measures on impulsivity and approach behavior.

Statistic	9–10 years old		11–12 years old		13–14 years old		15–16 years old		17–18 years old	
*n*	33		38		40		32		44	
Females/Males	10/23		17/21		19/21		15/17		27/17	
Age range (y;m)	8;8–10;10		11;0–12;11		13;0–14;11		15;0–16;11		17;0–18;11	
Mean age (y;m)	9;5		11;7		13;5		15;5		17;5	
SES (SD)	12.7	(2.4)	12.1	(2.3)	12.8	(2.7)	12.6	(2.2)	12.3	(2.7)
	*n* = 31		*n* = 34		*n* = 38		*n* = 30		*n* = 37	
Raven (SD)	24.9	(12.6)	33.3	(14.0)	38.6	(14.5)	50.4	(13.5)	53.0	(17.9)
Verbal Knowledge (SD)	35.7	(9.6)	48.8	(15.5)	55.8	(15.2)	67.1	(13.2)	69.8	(14.0)
IVE Impulsivity (SD)	8.4	(4.5)	7.2	(3.6)	7.7	(3.6)	6.4	(3.8)	7.0	(3.7)
IVE Venturesomeness (SD)	8.6	(3.9)	8.8	(4.7)	10.3	(3.3)	9.9	(4.3)	10.8	(3.5)
IVE Empathy (SD)	12.2	(3.5)	10.9	(4.7)	11.6	(3.1)	10.9	(3.7)	12.2	(3.2)
BAS (SD)	0.25	(0.75)	0.05	(0.71)	-0.18	(0.68)	-0.05	(0.82)	-0.02	(0.93)

*Scores on the Raven and Verbal Knowledge Tasks reflect percentage of correctly solved items. Possible range of values for all IVE subscales is 0–16. BAS composite scores reflect *z*-scores (standardized for the whole sample)*.

### Procedure

To assess decision-making in children and adolescents, we used four common decision-making contexts that are described in detail in the next section. Participants conducted the tasks in the context of a larger cross-sectional and longitudinal study to investigate the development of cognitive control and motivational functioning over the course of adolescence (age range = 9–18 years). The first measurement time T1 consisted of three sessions. In one session, participants received a comprehensive test battery, including cognitive tasks and the three decision-making tasks that lasted about 2–3 h. These tests and tasks were conducted on a computer using a 19-inch monitor, the computer keyboard, and a response box. In two further sessions, we collected electroencephalogram (EEG) data and measured task switching and reversal learning that will be reported elsewhere. Participants further completed various online self-report questionnaires conducted with the software program SoSci Survey (Leiner, 2014). These questionnaires collected information about, for example, demographic characteristics or traits such as reward responsiveness or impulsivity and were filled out at home between the sessions. The instructions of these questionnaires requested the children to fill out the questionnaire preferably undisturbed, but to ask their parents or the research team if problems occurred.

To keep motivation high, participants were told that their performance in the three decision-making tasks of the test battery were relevant to heighten the probability of winning a more valuable reward out of a box marked with three stars, rather than out of a one-star box, which were placed visibly for the participant in the laboratory. Unbeknown to the participants, all subjects received the feedback that they gained enough points to choose from the more valuable three-star box.

### Decision-Making Tasks

#### Treasure Hunting Task

**Figure 1:**
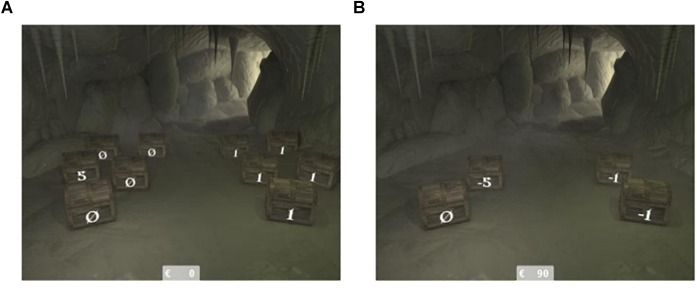
Illustration of the THT for the two valence domains, THT Gain **(A)** and THT Loss **(B)**, in which participants had to choose between a safe side (outcome is always the same) and a gamble between a high gain/loss or nothing, while all outcome probabilities are calculable.

This task is a modified version of the original CUPS tasks of Levin et al. (2007). In order to make it more child-friendly and to create a motivating context, we programmed a new version of this task, named THT, which is identical in the structure and conditions of the original CUPS task but different in task setting. As can be seen in Figure 1, the cups of the original task were replaced by treasure chests that were labeled with the containing number of 1€ coins. Like in the original CUPS task, participants were instructed to choose between a safe and a risky side, on which a varying number of treasure chests (2, 3, or 5) and its content (0 to 5 euros) were displayed (see Figure 1). Thereby, choosing the safe option always resulted in a sure gain or loss of 1€. Choosing the risky side resulted in either winning or losing a higher amount of money or winning or losing nothing. Risk-taking was measured as the percentage of risky side choices.

The experimental conditions consisted of two incentive values (gain or loss), three levels of expectancy (0.20, 0.33, or 0.50) by varying the number of cups on each side (2, 3, and 5), and three levels of outcome values for the risky side (2€, 3€, or 5€). In total, participants performed 54 trials. The trials were presented in a gain and a loss block, counterbalanced in order of presentation across participants in each age group. The other experimental conditions were randomized within each block. The blocks were further treated as separate task conditions, namely, as THT Gain and THT Loss conditions. In the THT Gain condition, gains were added to an account displayed on the lower screen starting from 0€, while in the THT Loss condition, losses were subtracted from the account starting from 90€ (see Figure 1). At the beginning of each block, participants conducted three practice trials to familiarize them with the task.

For the safe side, the expected outcome value (EV) was always 1€. For the risky side, three conditions in the gain and loss blocks resulted in an equal EV (0.20 × 5€, 0.33 × 3€, or 0.50 × 2€). Moreover, some combinations resulted in risk-advantageous EVs in which the EV of the risky option was more positive for gain (0.33 × 5€, 0.50 × 3€, or 0.50 × 5€) or less negative for loss trials (0.20 × 2€, 0.20 × 3€, or 0.33 × 2€) than the sure gain/loss of 1€. In other combinations, the EV was risk disadvantageous, as the EV of the risky option for these trials was less positive for the gain trials (0.20 × 2€, 0.20 × 3€, or 0.33 × 2€) or more negative for the loss trials (0.33 × 5€, 0.50 × 3€, or 0.50 × 5€) than the sure gain of the safe option. As we were mainly interested in a comparison of the three decision-making tasks in this study, we only used the overall percentage of risky side choices in the THT Loss and THT Gain conditions, respectively.

#### BART Task

**Figure 2:**
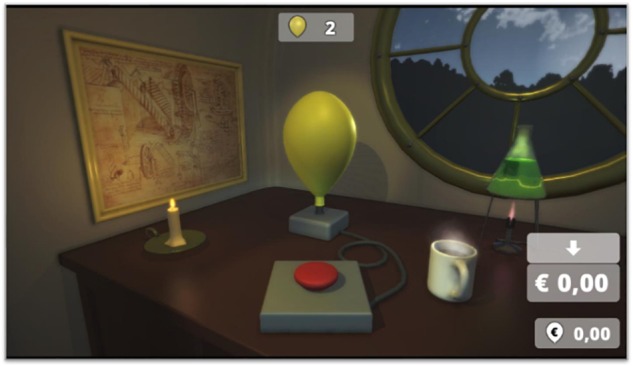
Illustration of the BART in which participants had to decide to inflate balloons, with each pump signifying an increase in respective outcome value but also in the risk for the balloon to burst and, thus, to lose all previous earnings.

In the BART (adapted from Lejuez et al., 2002), participants make decisions under increasing risk. They were instructed to inflate a virtual balloon with each pump signifying a temporal gain of 5 cents and the goal to collect as much money as possible. In this version, balloons could be inflated via a keypress activating a red button shown on the computer screen, which was visibly connected to the balloon (see Figure 2). The temporal gain of each balloon could be saved on a permanent “bank account” but would be lost if the balloon explodes before doing so. As the balloon could explode with any pump (probability of 1/128 for an explosion in first trial), participants had to weigh the increasing risk of the balloon to explode (probability of 1/128-n in the n-th trial) against the potential gain of pumping the balloon further. Therefore, risk-taking in the BART task was defined as the mean number of pumps taken, as more pumps signify a greater propensity for risk.

The task consisted of 30 balloons that were treated as separate trials and three practice trials, in which the participants were familiarized with the controls. During the task, participants had insight into how many of the 30 balloons are left, how much money was on their permanent bank account, and how much money they made with the previous balloon. Note that we again used the structure of the original BART task but changed the presentation of the balloon environment (see Figure 2). The BART was performed under two conditions: alone and under the observation of a fictitious peer. For the purpose of this study, we will include only the alone condition and used the mean number of pumps as dependent variable.

#### STOPLIGHT Task

**Figure 3:**
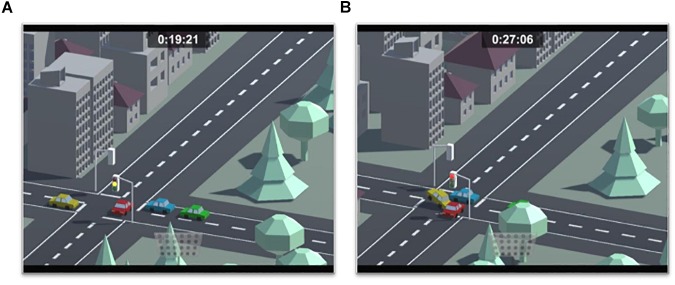
Illustration of the simulated driving task, the STOPLIGHT task, in which participants had to decide to break on or to overrun yellow traffic lights on a simulated track. Outcome options are either to lose a safe amount of time (STOP) or to gamble between losing no time (safe GO-decision, **A**) and causing an accident **(B)**.

The STOPLIGHT task (adapted from Chein et al., 2011) is a simulated driving task that has often been used as a behavioral measurement of risky decision-making. Again, we modified the original task to make the task environment similar to the other two decision-making tasks (see Figure 3). In this task, participants saw a car on a straight track from a bird’s eye view on a computer screen. Their goal was to reach a friend’s party as fast as possible. A timer on the upper screen counted time spent on the track visibly for the participant.

To progress on the track, participants had to advance through 20 intersections (10–16 s apart from one another) where a traffic light changed from green to yellow, as the vehicle approached. They had to decide whether to stop the otherwise automatically progressing car or to override the traffic light. To stop the car, participants had to press the space key of the computer keyboard in a time limit (2.5–4 s), which was indicated by an orange bar getting shorter as the car approached the intersection. Participants learned to control the car along the track through a tutorial that showed and instructed all three scenarios (stop at traffic light, override the traffic light without consequences, and causing a crash). At the end of the 20 intersections, which were treated as separate trials, participants arrived at the party that was animated in picture and sound. Stopping at the traffic light caused a time loss of 3 s. While overriding a yellow light could save the time else spent waiting, it also could cause a crash when the light changed to red, which resulted in an even bigger time loss of 6 s. The first 4 traffic lights were programmed to stay in the yellow phase, while the following 16 traffic lights changed from yellow to red in 50% of all cases. Risk-taking in the STOPLIGHT task was defined as the percentage of GO-decisions across all trials.

### Self-Report Measures on Impulsivity and Approach Behavior

#### Impulsiveness Questionnaire

We used the German adaption of the Impulsiveness Questionnaire I6 (IVE; Stadler et al., 2004) originally developed in English by Eysenck and Eysenck (1978). The IVE is a self-assessment questionnaire consisting of three subscales: impulsivity, venturesomeness, and empathy, with 16 items each. The subscales impulsivity and venturesomeness include items concerning cognitive and motivational impulsivity, as well as risk- and sensation-seeking behavior, while the subscale empathy inquires about the sensitivity for the feelings of others. The items consist of statements about the participant’s behavior (e.g., “Do you quite enjoy taking risks?”), which they could declare to be true (“yes”) or not (“no”). The authors provided data regarding the internal consistency of the German adaption with alpha coefficients ranging from 0.77 to 0.86. In this study, internal consistencies were 0.81, 0.84, and 0.84 for impulsivity, venturesomeness, and empathy, respectively.

#### BAS Scales

We used a translated version of the BAS scales (Carver and White, 1994) to assess approach behavior. The items were translated by members of our research team into child-friendly German. The BAS contains three subscales: reward responsiveness (five items), drive (four items), and fun seeking (four items). The items reflect statements (e.g., “When I want something, I usually go all out to get it”) that are answered via a four-point Likert scale, ranging from 1 (“strongly disagree”) to 4 (“strongly agree”). As subscales of the BAS were highly correlated (all *r*’s > 0.42), the *z*-standardized subscale scores were averaged. In this study, internal consistency for the BAS score reached an alpha coefficient of 0.80 for the whole sample.

### Fluid Intelligence and Control Variables

#### Advanced Progressive Matrices

To assess fluid intelligence, we used a computerized version of the Raven’s Advanced Progressive Matrices (APM; Raven et al., 1985). For time reasons, the test was time limited in our study, and participants had 15 min to solve the matrices. As scores, we used the percentage of correctly solved items during this time.

##### SES

The participants’ parents filled out a self-report questionnaire regarding socioeconomic information, family status, and health issues concerning the participating child. As these are the most widely used dimensions relevant for the SES, the highest education and highest occupation of the parents (cf. Nucci et al., 2012) as well as the monthly household net income were used to compute an SES score (Lampert et al., 2014). The SES was mainly used to describe our sample (see Table 1).

##### Verbal knowledge

To assess crystallized intelligence, we adapted two measures of verbal knowledge, the Word Puzzle and Word Similarities, of a German test for cognitive abilities for children and adolescents from 9 to 18 years (Kognitiver Fähigkeits-Test für 4. bis 12. Klassen, Revision: KFT 4-12+R, Heller and Perleth, 2000). Each task includes 12 words or word bundles, where participants either had to find the word with the same meaning (word puzzle) or they had to state which word would fit into specific word groups (word similarities). Each task ended after 4 min. As scores, we used the percentage of solved items in both tasks.

### Power Analyses

We conducted a *post hoc* power analysis with the program G^∗^Power (version 3.1, Faul et al., 2007) to find out whether our design had enough power to detect developmental trends in the four decision-making tasks. The analysis revealed that based on the means, standard deviation (SD), and correlation matrix of the four task settings, we would expect a large effect size in the within-between interaction (*f* = 1.49). Given our sample size (*N* = 187), the power of this effect to reach the 5% significance level was larger than 99%. For effect sizes as justified by Cohen, 1977; Cohen, 1988, we still obtained a power larger than 99% for a medium effect size of *f* = 0.25.

Concerning out hierarchical regression models, *post hoc* power analyses revealed a power of 69% to detect an *R*^2^ increase of 0.05. Such an increase in *R*^2^ was found for the four predictors of individual differences (BAS, impulsivity, venturesomeness, and empathy) when entered as the last step into the model including overall eight predictors for the THT Loss and STOPLIGHT. Given our sample of *N* = 187, we still obtained considerable power in detecting smaller effect sizes at the 5% significance level.

## Results

The present study examined the influence of age and individual differences in temperament components on four types of decision-making contexts. The Results section is structured along our main questions. First, we tested for differential age effects from late childhood to late adolescence on risk propensity of the four decision-making tasks. Second, we examined whether individual differences in temperament (i.e., approach behavior, impulsivity, venturesomeness, and empathy) can explain individual differences in risky decisions above and beyond age, gender, and fluid intelligence, and whether these influences differed depending on the task context. All analyses were conducted using SPSS (Version 24).

### Is There a Differential Influence of Age on Experimental Risk-Taking Contexts?

**Figure 4:**
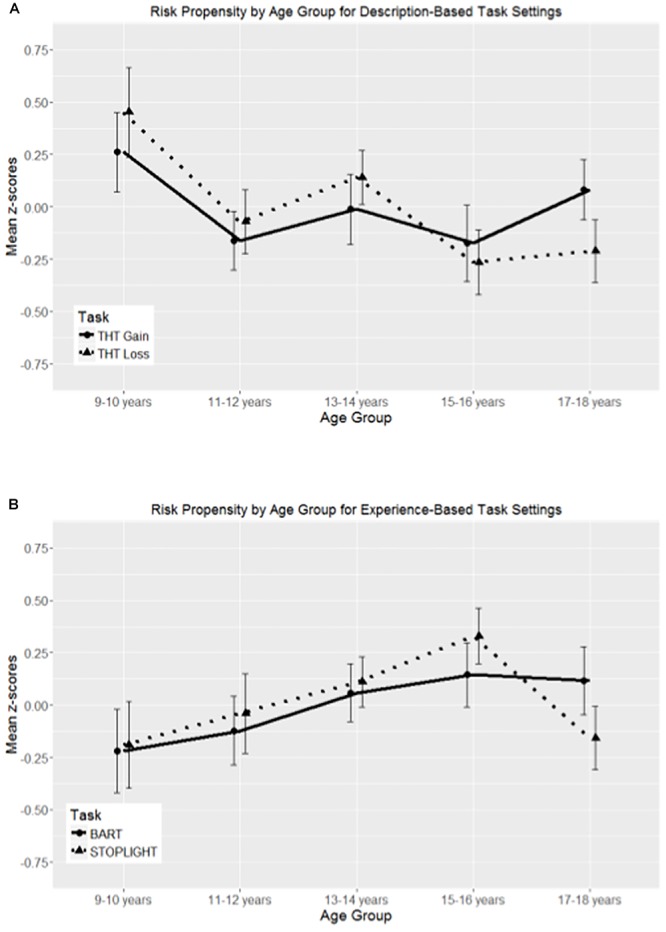
Mean *z*-scores of the four experimental decision-making tasks as a function of age group, presented separately for experience- **(A)** and description-based **(B)** task settings. Error bars represent standard errors. Points are offset horizontally so that error bars are visible.

To answer this question, we performed a multivariate analysis of variance (MANOVA) with age group (9–10, 11–12, 13–14, 15–16, and 17–18 years) as between-subjects variable and task types (THT Gain, THT Loss, BART, and STOPLIGHT) as dependent variables. For the within-factor task type, we predefined three contrasts: the first contrast compared mean differences in risk propensity between description-based and experience-based tasks, that is, between known and unknown outcome probabilities (contrasts: -1 -1 1 1). The second contrast determined the effect of valence for known outcome probabilities by comparing mean differences in risky decisions between gain and loss blocks of the THT (contrasts: -1 1 0 0). In the third contrast, we compared the mean of risky decisions between the BART and STOPLIGHT task (contrasts: 0 0 -1 1). For the between-factor age group, we contrasted for linear and quadratic age trends also in interaction with task type and furthermore tested for potential differences between age groups in a *post hoc* analysis. The corresponding data that entered into the analyses are shown in Table 2 as a function of task and age group, and mean *z*-scores for each task are displayed in Figure 4 as a function of age group.

**Table 2:** Percentage of Risky Decisions (SD) in the four risk-taking settings as a function of age group.

	THT Gain		THT Loss		BART		STOPLIGHT	
	*M*	*SD*	*M*	*SD*	*M*	*SD*	*M*	*SD*
9–10 years old	56.5	(16.0)	70.5	(17.7)	26.9	(13.6)	40.2	(19.7)
11–12 years old	50.2	(12.9)	63.0	(13.5)	28.0	(10.7)	42.6	(19.4)
13–14 years old	52.4	(15.8)	66.0	(11.8)	30.2	(10.4)	45.1	(12.5)
15–16 years old	50.0	(15.2)	60.2	(12.6)	31.2	(10.3)	48.7	(12.4)
17–18 years old	53.8	(13.7)	60.9	(14.3)	30.8	(12.8)	40.7	(16.6)

The results revealed a significant difference in risky decisions between description- and experience-based task settings, *F*(1,186) = 425.59, *p* < 0.001, η^2^ = 0.70, that was further modulated by a quadratic age trend, *F*(1,186) = 7.92, *p* < 0.01, η^2^ = 0.04. This finding indicated less risky decisions under unknown than known outcome probability and that this difference was less pronounced in mid-adolescents (see Table 2 and Figure 4A,B). We also obtained an effect of incentive valence, *F*(1,186) = 83.17, *p* < 0.001, η^2^ = 0.31, suggesting that more risky decision were taken in THT Loss than in THT Gain conditions (see Figure 4A). However, this effect was not further modulated by linear (only marginal) or quadratic age trends (*p* = 0.07 and *p* = 0.45, respectively). Finally, we also found a significant difference between the BART and STOPLIGHT, *F*(1,186) = 100.88, *p* < 0.001; η^2^ = 0.35, indicating more risky decisions in the STOPLIGHT (see Figure 4B). Again, this effect was not further modulated by linear or quadratic age trends *(p* = 0.40 and *p* = 0.12, respectively).

To better understand age differences, we performed multivariate age trend analysis for risk propensities in the tasks irrespective of task type. Thereby, age trend contrasts revealed a significant linear age effect in the THT Loss, *F*(1,182) = 8.97, *p* < 0.01, η^2^ = 0.05, suggesting a decrease in risky side choices over the course of adolescence. *Post hoc* comparisons using Bonferroni correction revealed that the 9- to 10-year-olds (*M* = 70.48, *SD* = 2.45) showed significantly more risky decisions than the 15- to 16-year-olds (*M* = 60.19, *SD* = 2.48) and the 17- to 18-year-olds (*M* = 60.94, *SD* = 2.12) in the THT Loss, while other age groups did not differ in their risk-taking (all *p*’s > 0.26). Moreover, no linear age effects were found for risk-taking in the THT Gain, the BART, or the STOPLIGHT (all *p*’s > 0.07). However, risky decisions in the STOPLIGHT suggested a quadratic age trend, *F*(1,182) = 4.00, *p* < 0.05; η^2^ = 0.02, that is, risk-taking was higher in mid-adolescents than in children and late adolescents. Thereby, *post hoc* comparisons using Bonferroni correction showed no differences in risk-taking between age groups in the STOPLIGHT (all *p*’s > 0.36).

### The Impact of Individual Differences in Temperament and Intelligence on Risk-Taking

At first, we analyzed correlations between sample characteristics and the outcome variables (the four risk-taking tasks) for the whole sample and the five age groups separately. As can be seen in Table 3, the correlations among the four risk-taking tasks are rather low and reached significance only for correlations between the two THT conditions (*r* = 0.31; *p* < 0.01) and the BART and STOPLIGHT (*r* = 0.15; *p* < 0.05). The pattern of results was quite similar for each of the five age groups. Therefore, separate hierarchical regression models were fitted for each of the four risk-taking contexts.

**Table 3:** Intercorrelations among the study variables.

Measure	1	2	3	4	5	6	7	8	9	10	11
1. THT Gain	—	0.31**	0.13	0.13	-0.02	-0.02	-0.10	0.14	0.12	0.02	0.15*
2. THT Loss	0.31**	—	0.13	0.12	-0.12	-0.06	-0.17*	0.15*	0.23**	0.03	0.12
3. BART	0.12	0.10	—	0.15*	0.05	-0.12	0.08	0.06	0.16*	-0.02	0.06
4. STOPLIGHT	0.13	0.11	0.15*	—	0.07	-0.02	-0.02	0.16*	0.14	0.07	0.09
5. Age	-0.04	-0.22**	0.13	0.05	—	0.08	0.02	-0.04	0.04	0.11	0.00
6. Gender	-0.03	-0.10	-0.08	-0.01	0.20**	—	-0.04	-0.08	-0.15*	0.40**	-0.13
7. Intelligence	-0.10	-0.25**	0.14	0.01	0.56**	0.08	—	-0.30**	-0.11	0.02	-0.07
8. Impulsivity	0.14	0.17*	0.05	0.15*	-0.12	-0.10	-0.32**	—	0.37**	-0.23**	0.35**
9. Venturesomeness	0.11	0.18*	0.18*	0.15*	0.20**	-0.11	0.02	0.34**	—	-0.08	0.35**
10. Empathy	0.02	0.03	-0.02	0.07	0.04	0.40**	0.03	-0.23**	-0.07	—	-0.03
11. BAS	0.15*	0.14	0.04	0.08	-0.10	-0.15*	-0.11	0.36**	0.33**	-0.03	—

*Intercorrelations with age group partialed out are presented above the diagonal, and intercorrelations for the whole sample are presented below the diagonal. THT Gain, THT Loss, BART, and STOPLIGHT are experimental decision-making tasks. Gender was categorized in 1 for male and 2 for female participants. Intelligence is a measure of fluid intelligence that derive from the Raven’s Progressive Matrices. Impulsivity, venturesomeness, empathy, and BAS are measures of individual differences in temperament that derive from the IVE (German version) and the BAS, respectively. ^∗^*p* < 0.05. ^∗∗^*p* < 0.01*.

For each regression model, we first entered age and age^2^ as we were interested in the explained variance beyond age effects. To reduce multicollinearity, age was centralized on the sample mean. In the next step, we entered gender and fluid intelligence to examine whether gender and individual differences in fluid abilities can explain some of the variance in risky decisions in the four task settings. In the final step, we entered the temperament measures (approach behavior, impulsivity, venturesomeness, and empathy) to examine their contribution in predicting risky behavior above age, gender, and development in fluid intelligence. Tests to see if the data met the assumption of collinearity indicated that multicollinearity was not a concern (age, tolerance = 1.00, VIF = 1.00; age^2^, tolerance = 1.00, VIF = 1.01; gender, tolerance = 0.96, VIF = 1.04; intelligence, tolerance = 0.68, VIF = 1.47; impulsivity, tolerance = 0.69, VIF = 1.45; venturesomeness, tolerance = 0.74, VIF = 1.35; empathy, tolerance = 0.77, VIF = 1.31; BAS, tolerance = 0.78, VIF = 1.28).

For the THT Gain and the BART task, neither the predictor variables nor the overall model reached significance (see Table 4). In contrast, a significant regression equation was found for risk decisions of the THT Loss condition, *F*(8,176) = 3.28, *p* < 0.01, with an *R*^2^ of 0.130. Adding age on the first step resulted in a significant increase in *R*^2^, *R*^2^ change = 0.050, *F*(1,183) = 9.56, *p* < 0.01. This partial effect of age (β = -0.22, *p* < 0.01) was further superseded by the effect of fluid intelligence (β = -0.20, *p* < 0.05) when added to the model, without increasing *R*^2^ further, *R*^2^ change = 0.028, *F*(4,180) = 3.89, *p* < 0.01. In the final model, there was a significant relationship between risk-taking propensity in the THT Loss condition and the venturesomeness subscale (β = 0.20, *p* < 0.05), with the final step including individual differences in temperament generally increasing the model fit significantly, *R*^2^ change = 0.050, *F*(8,176) = 3.28, *p* < 0.01.

**Table 4:** Results of the stepwise regression analysis for the four risk-taking settings.

	Source of risk-taking behavior							
	THT Gain		THT Loss		BART		STOPLIGHT	
Predictor	*R*^2^	β	*R*^2^	β	*R*^2^	β	*R*^2^	β
**Step 1**	0.002		0.050 **		0.018†		0.003	
Age		-0.040		-0.223**		0.133†		0.051
**Step 2**	0.003		0.002		0.008		0.033*	
Age^2^		0.051		-0.039		-0.088		-0.182*
**Step 3**	0.010		0.028†		0.018		0.001	
Gender		-0.030		-0.058		-0.118		-0.024
Intelligence		-0.119		-0.195*		0.080		-0.035
**Step 4**	0.029		0.050*		0.023		0.055*	
Impulsivity		0.076		0.049		0.040		0.171*
Venturesomeness		0.062		0.195*		0.134		0.055
Empathy		0.054		0.087		0.059		0.180*
BAS		0.091		0.017		-0.003		0.027
**Total *R*^2^ n**	0.043		0.130**		0.067		0.091*	
	185		185		185		185	

*Intelligence is a measure of fluid intelligence that derive from the Raven’s Progressive Matrices. Gender was categorized in 1 for male and 2 for female participants. Impulsivity, venturesomeness, empathy, and BAS are measures of individual differences in temperament that derive from the IVE (German version) and the BAS, respectively. ^†^*p* < 0.10. ^∗^*p* < 0.05. ^∗∗^*p* < 0.01*.

Furthermore, also 9% of the variance of risky decisions in the STOPLIGHT task could be explained by the final regression model, *R*^2^ = 0.091 [*F*(8,176) = 2.21, *p* < 0.05]. Thereby, age^2^ (β = -0.20, *p* < 0.01), impulsivity (β = 0.17, *p* < 0.05), and empathy (β = 0.18, *p* < 0.05) showed significant partial effects, with *R*^2^ change = 0.033, *F*(2,182) = 3.34, *p* < 0.01 for the step including age^2^ and *R*^2^ change = 0.055, *F*(8,176) = 2.21, *p* < 0.05 for the step including the measures of individual differences in temperament, respectively.

## Discussion

The main goals of this study were to determine age differences in different decision-making tasks across a broad age range throughout adolescence and to explore the role of individual differences in temperament in understanding age differences in decision-making. Therefore, we applied four experimental decision-making tasks (THT Gain and Loss, BART, and STOPLIGHT) to analyze developmental trends from late childhood to late adolescence. The tasks differed in task characteristics, such as known outcome probability, valence of anticipated outcomes (i.e., gains and losses), dynamic change of risk level, and induced time pressure. Additionally, we were interested in the (possibly differential) contribution of individual differences in temperament components, namely, approach behavior, impulsivity, venturesomeness, and empathy, in explaining risky decisions.

The results of our study revealed several important new insights. At first, the four decision-making tasks indeed showed differential developmental patterns throughout adolescence. Second, the experimental risk-taking tasks were only low to moderately correlated with each other, indicating that each of them captures a unique decision-making context. Moreover, only some decision-making were susceptible to individual differences in temperament and fluid intelligence.

Considering first decision-making in a loss context, results of our study clearly indicated that gambling to prevent losses diminished with increasing age for task settings under known outcome probabilities (THT Loss). This is in line with a recent study that compared decision-making separately for the gain and loss domains (van den Bos and Hertwig, 2017). According to Reyna and Farley (2006), an increasing risk aversion with age can be explained by a developmental shift from basing decisions on quantitative to qualitative outcome dimensions (e.g., preferring to possibly lose nothing than to lose something) over the course of adolescence. In contrast, results of our study indicated that decision-making under gain conditions was not age sensitive, while other studies revealed a mid-adolescent peak in reaction to potential gains (e.g., van den Bos and Hertwig, 2017), or at least a small increase in risk propensity with age (aged 8–17 years; Levin et al., 2014). A possible explanation for these contradicting findings might be differential sensitivities to gains and losses depending on the value of potential outcomes across adolescence. As such, according to our findings, adolescents showed more risk-seeking behavior in the loss than in the gain domain (aged 14–17 years; Reyna et al., 2011), at least under the prospect of small to medium incentives ($5 and $20). Thus, in high-reward conditions ($150), adolescents showed a reversed framing effect with more risky decisions in the gain than in the loss condition (Reyna et al., 2011). Hence, in line with a recent review, a “hot” context, like when high incentive values are given, more consistently provoke an adolescent-specific reaction (cf. Kray et al., 2018). Moreover, van den Bos and Hertwig (2017) showed an inverted U shape in risk propensities for gain gambles under known risk across adolescence but also used a higher variability in incentive values (from 3€ to 32€) than we offered in the THT (2€, 3€, or 5€). This is also in line with the prospect theory that considers differential reference points, like incentive domain and value range, in the prediction of risky decision-making behavior. Thus, the development in processing multiple outcome characteristics, like referencing actual outcome with respect to the maximal earnable value, might further explain age differences in risky decisions.

In support of this view, we found not only decreasing risk-taking to prevent losses with age but also that this decreasing can mostly be accounted for by individual differences in fluid intelligence. In a previous study, individual differences in cognitive abilities, like numeracy, have also been associated with a higher sensitivity for expected values and thus more advantageous risk decisions with development (Levin et al., 2014). While adolescents have been shown to be capable decision-maker in age-appropriate and coherent decision situations (e.g., Crone et al., 2003), it may be that the level of information in the THT Loss was too demanding for the still immature cognitive abilities of children and young adolescents. Beneath individual differences in cognitive abilities, more risky decisions in the THT Loss were associated with a higher degree of self-reported venturesomeness. Venturesomeness is thereby the motivation to explore risk behaviors for which participants are aware of potential risks. Reyna and Farley (2006) described a similar phenomenon in risk preferences of youth, showing that even though adolescents tend to overestimate the true likelihood of negative outcomes of risk behaviors (e.g., HIV), they engage in heightened risk-taking (e.g., unprotected sex; Reyna and Farley, 2006). Importantly, the influence of individual differences in venturesomeness remained significant even after controlling for individual differences in age and thus may explain motivation to engage in known risks above adolescent development. However, an open question for future research remains whether influences of both individual differences in fluid intelligence and venturesomeness are adaptive or maladaptive in risky decision-making, that is, whether increasing risk aversion with fluid intelligence and/or the disposition to explore risk options will lead to more risk-advantageous choices or even to worse performance (choices for risk-disadvantageous options) in decisions to prevent losses with known probabilities. While our temperament measures generally increased the predictability of risk decisions in the THT Loss, no other individual difference except for venturesomeness predicted risk decisions in the THT Loss significantly. It has already been argued that task-based risk measures, like the THT, might reflect a different behavioral manifestation of risk-taking than risk propensity (e.g., self-reported reward sensitivity) and frequency measures (real-life risk behaviors, e.g., drinking). Nonetheless, task contexts might reflect states for which certain individual temperamental differences predict risk decisions more reliable than others (Frey et al., 2017). Thus, the tendency to engage in known risks (venturesomeness), for example, might rather reflect risk decisions under described potential losses but not gains.

Thus, and in accordance with several findings for the gain domain under known risk (for BAS, Blankenstein et al., 2018; for novelty-/thrill-seeking, van den Bos and Hertwig, 2017), risk propensity in the THT Gain cannot be predicted by any of the given individual differences. The lack of an association between risk-taking in the THT Gain and fluid intelligence is surprising, however. In contrast, risk propensity in the THT Loss and the sensitivity to expected values across valence domains of the CUPS task could completely be accounted by fluid intelligence or numeracy abilities (age range = 8–17 years; Levin et al., 2014), respectively. Thus, in an earlier study, Levin et al. (2007) could show that EV sensitivity had a more protracted development in the loss than in the gain domain of the CUPS, at least when compared between younger and older children (aged 5–7 and 8–11 years, respectively). Generally, it has been shown that resources are differentially involved in the processing of positive versus negative information in a variety of psychological processes, for which all losses have a higher impact (for a review, see Baumeister et al., 2001). Thus, they might allocate more cognitive resources than gains.

For dynamic risk conditions, such as choices to pump the balloon in the BART, we found no age sensitivity in the present study. Given the fewer risk decisions in experience- than description-based task settings, a lack of age differences in the BART matches the finding of Van Duijvenvoorde et al. (2012). They could show that participants under age 12 could not learn from experience at all during experimental decision-making, while learning from described outcomes was already present in late childhood. In addition, other studies reported that risk propensity seems to rather peak in late adolescence or young adulthood with a decline thereafter (Braams et al., 2015; Duell et al., 2018), hence a U-shaped developmental trend when including also young adults. However, as our sample did not include age groups above age 18, we might not be able to depict the plateau and consecutive decline of risk-taking in the BART. Given that this study is designed as a longitudinal study with a lag of 2 years, we might be able to obtain similar developmental trends as reported in the future. However, decisions for risky options under time pressure in the STOPLIGHT showed the hypothesized mid-adolescent peak, which is in line with previous findings (Steinberg et al., 2008; Duell et al., 2018). Moreover, adolescents engaged in higher risk in the STOPLIGHT than in the BART, which is in line with the finding of relatively risk-averse behavior in the BART with respect to the maximal number of possible consecutive risk decisions and the tasks’ maximized point earnings (for a review, see Lauriola et al., 2014). As such, decisions to engage in risks when dynamic probabilities are only experienced might increase with rather protracted task and and/or life experience, as compared to tasks with static risk, like the STOPLIGHT.

Accordingly, the two experienced-based tasks showed different susceptibility to individual differences in temperament. Our regression model revealed that neither age nor gender, fluid intelligence, and temperamental differences did explain risk behavior in the BART. As the most profound correlations between risk propensity in the BART and temperamental differences in approach behavior and disinhibition seem to rise with age (BAS Drive, Braams et al., 2015; sensation seeking and impulsivity, Lauriola et al., 2014; for the BART-Y, MacPherson et al., 2010), here again, the chosen age range might not be not optimal to depict these associations. For the STOPLIGHT, however, our regression model indicated that, above age, gender, and fluid intelligence, two temperamental facets, namely, impulsivity and empathy, predicted risky behavior in the STOPLIGHT. Thereby, other studies did not find an association between risk propensity in the STOPLIGHT and impulsivity, as measured by the Barratt Impulsiveness Scale (BIS-11, Barratt, 1959), but with sensation seeking (Steinberg et al., 2008; Chein et al., 2011). Sensation seeking (Zuckerman, 2007) is thereby a measure for thrill-seeking tendencies with some overlap to the IVE subscale venturesomeness used in this study. However, these studies investigated older samples, and it has to be acknowledged that the BIS-11 was not conceptualized for children and younger adolescent samples, as it includes items that might not reflect impulsive behavior appropriate for these ages (e.g., “I spend more money than I earn”). Therefore, we applied the IVE in the present study as it showed sufficient validity and its impulsivity measure is adapted for younger samples. Hence, differences in sample characteristics and measurement instruments may explain the differences in outcomes. Interestingly, social context manipulations, like an observation by peers while performing the STOPLIGHT, have been shown to induce more risky decisions in adolescents (aged 14–18 years) but for no other age group (aged 19–22 years and 24–29 years, respectively; Chein et al., 2011). Similarly, in this study, the proportion of risky decisions can be explained by individual differences in a measure of social sensitivity, namely, empathy. Here, more empathic participants showed more risky behavior in the STOPLIGHT. One explanation might be that those participants that are empathic for the feelings of others are also those that feel rewarded to engage in a risk that has potential consequences for (accident) or is seen (virtual traffic or peers) by others. Thereby, it has to be acknowledged that we changed the visual environment of these tasks to make them dynamic and appealing in use for early to late adolescents. While we intended to maximize the affective context, participants could evaluate negative outcomes, thus accidents as less severe when seen from bird’s eye view in a rather plastic surrounding like in this study (see Figure 3). This could account for the positive direction of the association between risk-taking in the STOPLIGHT and empathy. Another explanation might be on the side of the time pressure manipulation, as participants were asked to reach a friend’s party in a timely fashion while being already late during the STOPLIGHT. Thus, empathic participants might be more driven and more willing to engage in risk to reach this goal to not be displeasing.

### Limitations of the Present Findings

A limitation that can be drawn on most studies using experimental decision-making is that their relevance in explaining real-life risk behaviors in adolescence remains unclear. As such, even though we can show that several affective task moderators influence decision-making in the laboratory, we cannot conclude their meaning for decisions to engage in health-risk behaviors across adolescence. Generally, a study using psychometric modeling analyses found that self-reported behavioral tendencies in risky decision-making were more related to frequencies of real-life risk behavior (like alcohol or cigarette consumption) than risky choices in experimental tasks. Moreover, self-reported risk preferences appear to be more stable over time than experimental risk measures, which are thought to rather capture states than traits (Frey et al., 2017). The fact that quite variable and often undefined personality measures are used in the decision-making literature and often quite low sample sizes to detect associations between individual differences and task performance may further contribute to the difference between experimental and self-descriptive measures (Appelt et al., 2011). Yet, each behavioral task represents a specific choice frame that can be used to examine inter- and intraindividual differences in reaction to these decision contexts (Frey et al., 2017).

Moreover, to compare task settings that differ in affectively engaging task moderators, we implemented one representative of each decision-making context we were interested in. This leads to a main limitation in generalizing our findings to the numerous experimental risky decision-making tasks found in the literature. Specifically, the actual findings further emphasize to consider the role of affective contexts and individual differences in fluid intelligence and temperament instead of generalizing risk-taking behavior in adolescence. Nonetheless, the task settings used are counted among the most investigated experimental decision-making tasks in the adolescent literature and showed benefits in evoking specific affective states.

In this study, we found adolescent risky decision-making and the predictive value of their individual temperamental differences to be context dependent. Thereby, a main limitation, so far, is the reliance on only few age groups and tasks when investigating developmental trends in adolescent decision-making. Even though we overcame this limitation and made use of the full age range from early to late adolescence, age was not the most decisive predictor of experimental risk-taking. Thereby, literature drawing conclusions between motivated decision-making and pubertal development in adolescence is growing (for a review, see Laube and van den Bos, 2016). However, self-description measures of pubertal status often are closely related to age. This makes the comparison between influences of pubertal status and close age groups, without the intention to measure hormone levels in blood or salivary, difficult. Even though we draw our conclusions based on a wide age range and variable task contexts, cross-sectional data remain inferior to longitudinal data when detecting changes over time or individual pubertal development. As our findings derive from our first measurement period, which will be followed by a second measuring point within a 2-year gap, we can use the gathered information about the given task settings and their sensitivity to individual differences to formulate more specific predictions concerning changes in risk propensity over time.

## Conclusion and Outlook

In conclusion, results of this study revealed that risk propensity across adolescence is highly context dependent. More specifically, while risk-taking propensity showed an adolescent-specific peak for experienced task settings under time pressure (STOPLIGHT), it declined with increasing cognitive abilities in gambles to prevent losses with known outcome probabilities (THT Loss).

For the comparison of the gain and loss domains under known outcome probabilities, the gain domain of the THT was not age sensitive in this study, and our measure of reward sensitivity (BAS) could not explain variance of any risk-taking measure. Adolescents moreover were more risk seeking when deciding between options to minimize risks than to maximize gains. Nonetheless, most findings in the adolescent literature are limited to gains, even though social contexts have been shown to have a high impact on decision-making in adolescence, with only few decision-making tasks being investigated under social context manipulations so far. In sum, one should consider the age-specific relevance of different kinds of contexts and incentives when exploring the impact of reward/punishment sensitivity on risk-taking behavior from childhood to adulthood (see Kray et al., 2018 for a review).

For the comparison of experience-based versus description-based outcome probabilities, adolescents engage in more risky decisions when outcome probabilities are known than unknown. In addition, description-based tasks in the loss domain are associated with more deliberate functioning (fluid intelligence and venturesomeness), while experience-based task settings under time pressure are rather associated with affective functioning (impulsivity and empathy). This finding underlines the importance to distinguish disinhibition behavior associated with more cognitive (to engage in known risks, venturesomeness) or more affective functioning (to act without thinking, impulsivity) (see Eysenck and Eysenck, 1978). Moreover, risk aversion in experience-based decision-making was higher when risk probability changed dynamically with each decision in the BART. In sum, the results of our study indicate adolescent risky decisions to be context dependent and differentially susceptible to individual temperamental differences in experimental decision-making settings with described as well as experienced outcome probabilities.

## Ethics Statement

This study was carried out in accordance with the recommendations of the Declaration of Helsinki and approved by the local ethics committee of Saarland University. All subjects gave written informed consent and were paid 8€ per hour.

## Author Contributions

CL was responsible for the content of this original study. JK provided feedback regarding the analysis, interpretation and discussion, and gave advice in the writing process.

## Conflict of Interest Statement

The authors declare that the research was conducted in the absence of any commercial or financial relationships that could be construed as a potential conflict of interest.

## References

[B1] Allemand M. Steiger A. E. Fend H. A. (2015). Empathy development in adolescence predicts social competencies in adulthood. *J. Pers.* 83 229–241. 10.1111/jopy.12098 24684661

[B2] Appelt K. C. Milch K. F. Handgraaf M. J. J. Weber E. U. (2011). The Decision Making Individual Differences Inventory and guidelines for the study of individual differences in judgment and decision-making research. *Judgm. Decis. Mak.* 6 252–262.

[B3] Barberis N. C. (2013). Thirty Years of Prospect Theory in Economics: A Review and Assessment. *J. Econ. Perspect.* 27 173–196. 10.1257/jep.27.1.173

[B4] Barratt E. S. (1959). Anxiety and impulsiveness related to psychomotor efficiency. *Percept. Mot. Skills* 9 191–198. 10.2466/pms.1959.9.3.191

[B5] Baumeister R. F. Bratslavsky E. Finkenauer C. Vohs K. D. (2001). Bad is stronger than good. *Rev. Gen. Psychol.* 5 323–370. 10.1037//1089-2680.5.4.323

[B6] Benjamin D. J. Shapiro J. M. (2005). *Does Cognitive Ability Reduce Psychological Bias?*. *JEL Manuscript J24* (accessed May 16 2007).

[B7] Blakemore S.-J. (2012). Development of the social brain in adolescence. *J. Royal Soc. Med.* 105 111–116. 10.1258/jrsm.2011.110221 22434810 PMC3308644

[B8] Blakemore S.-J. Mills K. L. (2014). Is adolescence a sensitive period for sociocultural processing? *Ann. Rev. Psychol.* 65 187–207. 10.1146/annurev-psych-010213-115202 24016274

[B9] Blankenstein N. E. Schreuders E. Peper J. S. Crone E. A. van Duijvenvoorde A. C. K. (2018). Individual differences in risk-taking tendencies modulate the neural processing of risky and ambiguous decision-making in adolescence. *Neuroimage* 172 663–673. 10.1016/j.neuroimage.2018.01.085 29408323

[B10] Braams B. R. van Duijvenvoorde A. C. K. Peper J. S. Crone E. A. (2015). Longitudinal changes in adolescent risk-taking: a comprehensive study of neural responses to rewards, pubertal development, and risk-taking behavior. *J. Neurosci.* 35 7226–7238. 10.1523/JNEUROSCI.4764-14.2015 25948271 PMC6605271

[B11] Byrnes J. P. Miller D. C. Schafer W. D. (1999). Gender differences in risk taking: a meta-analysis. *Psychol. Bull.* 125 367–383. 10.1037/0033-2909.125.3.367

[B12] Carver C. S. White T. L. (1994). Behavioral inhibition, behavioral activation, and affective responses to impending reward and punishment: the BIS/BAS scales. *J. Pers. Soc. Psychol.* 67 319–333. 10.1037/0022-3514.67.2.319

[B13] Casey B. J. Jones R. M. Hare T. A. (2008). The adolescent brain. *Ann. N. Y. Acad. Sci.* 1124 111–126. 10.1196/annals.1440.010 18400927 PMC2475802

[B14] Cazzell M. Li L. Lin Z. J. Patel S. J. Liu H. (2012). Comparison of neural correlates of risk decision making between genders: an exploratory fNIRS study of the Balloon Analogue Risk Task (BART). *Neuroimage* 62 1896–1911. 10.1016/j.neuroimage.2012.05.030 22634214

[B15] Chein J. Albert D. O’Brien L. Uckert K. Steinberg L. (2011). Peers increase adolescent risk taking by enhancing activity in the brain’s reward circuitry. *Dev. Sci.* 14 F1–F10. 10.1111/j.1467-7687.2010.01035.x 21499511 PMC3075496

[B16] Collado A. Felton J. J. W. MacPherson L. Lejuez C. C. W. (2014). Longitudinal trajectories of sensation seeking, risk taking propensity, and impulsivity across early to middle adolescence. *Addict. Behav.* 39 1580–1588. 10.1016/j.addbeh.2014.01.024 24566195 PMC4117709

[B17] Crone E. A. Vendel I. van der Molen M. W. (2003). Decision-making in disinhibited adolescents and adults: insensitivity to future consequences or driven by immediate reward? *Pers. Individ. Dif.* 34 1–17.

[B18] Crone E. A. Dahl R. E. (2012). Understanding adolescence as a period of social–affective engagement and goal flexibility. *Nat. Rev. Neurosci.* 13 636–650. 10.1038/nrn3313 22903221

[B19] Dahl R. E. (2004). Adolescent brain development: a period of vulnerabilities and opportunities. *keynote address*. *Ann. N. Y. Acad. Sci.* 1021 1–22. 10.1196/annals.1308.001 15251869

[B20] Defoe I. N. Dubas J. S. Figner B. van Aken M. A. G. (2015). A meta-analysis on age differences in risky decision-making: adolescents versus children and adults. *Psychol. Bull.* 141 48–84. 10.1037/a0038088 25365761

[B21] Dick B. Ferguson B. J. (2015). Health for the world’s adolescents: a second chance in the second decade. *J. Adolesc. Health* 56 3–6. 10.1016/j.jadohealth.2014.10.260 25530601

[B22] Donkers B. Melenberg B. Van Soest A. (2001). Estimating risk attitudes using lotteries: a large sample approach. *J. Risk Uncertain.* 22 165–195.

[B23] Duell N. Steinberg L. Chein J. Al-Hassan S. M. Bacchini D. Lei C. (2016). Interaction of reward seeking and self-regulation in the prediction of risk taking: a cross-national test of the dual systems model. *Dev. Psychol.* 52 1593–1605. 10.1037/dev0000152 27598251

[B24] Duell N. Steinberg L. Icenogle G. Chein J. Chaudhary N. Di Giunta L. (2018). Age patterns in risk taking across the world. *J. Youth Adolesc.* 47 1052–1072. 10.1007/s10964-017-0752-y 29047004 PMC5878702

[B25] Ernst M. (2014). The triadic model perspective for the study of adolescent motivated behavior. *Brain Cogn.* 89 104–111. 10.1016/j.bandc.2014.01.006 24556507 PMC4248307

[B26] Eysenck S. B. Eysenck H. J. (1978). Impulsiveness and venturesomeness: their position in a dimensional system of personality description. *Psychol. Rep* 43 1247–1255. 10.2466/pr0.1978.43.3f.1247 746091

[B27] Faul F. Erdfelder E. Lang A.-G. Buchner A. (2007). G^∗^Power 3: A flexible statistical power analysis program for the social, behavioral, and biomedical sciences. *Behav. Res. Methods* 39 175–191. 10.3758/BF03193146 17695343

[B28] Figner B. Mackinlay R. J. Wilkening F. Weber E. U. (2009). Affective and deliberative processes in risky choice: age differences in risk taking in the Columbia Card Task. *J. Exp. Psychol. Learn. Mem. Cogn.* 35 709–730. 10.1037/a0014983 19379045

[B29] Frey R. Pedroni A. Mata R. Rieskamp J. Hertwig R. (2017). Risk preference shares the psychometric structure of major psychological traits. *Sci. Adv.* 3:e1701381. 10.1126/sciadv.1701381 28983511 PMC5627985

[B30] Galván A. McGlennen K. M. (2012). Daily stress increases risky decision-making in adolescents: a preliminary study. *Dev. Psychobiol.* 54 433–440. 10.1002/dev.20602 22012864

[B31] Galván A. Rahdar A. (2013). The neurobiological effects of stress on adolescent decision making. *Neuroscience* 249 223–231. 10.1016/j.neuroscience.2012.09.074 23069759

[B32] Giedd J. N. Blumenthal J. Jeffries N. O. Castellanos F. X. Liu H. Zijdenbos A. (1999). Brain development during childhood and adolescence: a longitudinal MRI study. *Nat. Neurosci.* 2 861–863. 10.1038/13158 10491603

[B33] Gray J. A. (1972). “The psychophysiological basis of introversion–extraversion: a modification of Eysenck’s theory,” in *The Biological bases of Individual Behaviour*, eds Nebylitsyn V. D. Gray J. A. (New York: Academic Press), 182–205. 10.1016/b978-0-12-515350-8.50017-x

[B34] Harris C. R. Jenkins M. Glaser D. (2006). Gender differences in risk assessment: why do women take fewer risks than men? *Judgm. Decis. Mak.* 1 48–63. 11398025

[B35] Heller K. A. Perleth C. (2000). *Kognitiver Fähigkeitstest Für 4. bis* 12 Klassen, Revision: KFT 4-12+ R. Bad Langensalza: Beltz-Test.

[B36] Hertwig R. Barron G. Weber E. U. Erev I. (2004). Decisions from experience and the effect of rare events in risky choice. *Psychol. Sci.* 15 534–539. 10.1111/j.0956-7976.2004.00715.x 15270998

[B37] Hertwig R. Erev I. (2009). The description–experience gap in risky choice. *Trends Cogn. Sci.* 13 517–523. 10.1016/j.tics.2009.09.004 19836292

[B38] Kahneman D. Tversky A. (1979). Prospect theory: an analysis of decisions under risk. *Econometrica* 47 263–291. 10.2307/1914185

[B39] Kim-Spoon J. Kahn R. Deater-Deckard K. Chiu P. Steinberg L. King-Casas B. (2016). Risky decision making in a laboratory driving task is associated with health risk behaviors during late adolescence but not adulthood. *Int. J. Behav. Dev.* 40 58–63. 10.1177/0165025415577825 26770006 PMC4707653

[B40] Knyazev G. G. Slobodskaya H. R. Wilson G. D. (2004). Comparison of the construct validity of the Gray–Wilson Personality Questionnaire and the BIS/BAS scales. *Pers. Individ. Dif.* 37 1565–1582. 10.1016/j.paid.2004.02.013

[B41] Kray J. Schmitt H. Lorenz C. Ferdinand N. K. (2018). The influence of different kinds of incentives on decision-making and cognitive control in adolescent development: a review of behavioral and neuroscientific studies. *Front. Psychol.* 9:768. 10.3389/fpsyg.2018.00768 29875720 PMC5974121

[B42] Lampert T. Müters S. Stolzenberg H. Kroll L. E. (2014). Messung des sozioökonomischen Status in der KiGGS-Studie. *Bundesgesundheitsblatt Gesundheitsforschung Gesundheitsschutz* 57 762–770. 10.1007/s00103-014-1974-8 24950825

[B43] Laube C. van den Bos W. (2016). Hormones and affect in adolescent decision-making. *Recent Dev. Neurosci. Res. Hum. Motiv* 19 259–281. 10.1108/S0749-742320160000019013

[B44] Lauriola M. Panno A. Levin I. P. Lejuez C. W. (2014). Individual differences in risky decision making: a meta-analysis of sensation seeking and impulsivity with the Balloon Analogue Risk Task. *J. Behav. Dec. Making* 27 20–36. 10.1002/bdm.1784

[B45] Leiner D. J. (2014). *SoSci Survey (Version 2.5.00-i) [Computer Software]*. Available at: http://www.soscisurvey.com (accessed July 1 2019).

[B46] Lejuez C. W. Aklin W. M. Zvolensky M. J. Pedulla C. M. (2003). Evaluation of the Balloon Analogue Risk Task (BART) as a predictor of adolescent real-world risk-taking behaviours. *J. Adolesc.* 26 475–479. 10.1016/S0140-1971(03)00036-8 12887935

[B47] Lejuez C. W. Read J. P. Kahler C. W. Richards J. B. Ramsey S. E. Stuart G. L. (2002). Evaluation of a behavioral measure of risk taking: the Balloon Analogue Risk Task (BART). *J. Exp Psychol. Appl.* 8 75–84. 10.1037//1076-898X.8.2.75 12075692

[B48] Levin I. P. Bossard E. A. Gaeth G. J. Yan H. (2014). The combined role of task, child’s age and individual differences in understanding decision processes. *Judgm. Decis. Mak.* 9 273–285.

[B49] Levin I. P. Weller J. A. Pederson A. Harshman L. (2007). Age-related differences in adaptive decision making: sensitivity to expected value in risky choice. *Judgm. Decis. Mak.* 2 225–233.

[B50] Li S. Lindenberger U. Hommel B. Aschersleben G. Prinz W. Baltes P. B. (2004). Transformations in the couplings among intellectual abilities and constituent cognitive processes across the life span. *Psychol. Sci.* 15 155–163. 10.1111/j.0956-7976.2004.01503003.x 15016286

[B51] Loxton N. J. Dawe S. (2001). Alcohol abuse and dysfunctional eating in adolescent girls: the influence of individual differences in sensitivity to reward and punishment. *Int. J. Eat. Disord.* 29 455–462. 10.1002/eat.1042 11285583

[B52] Luna B. Garver K. E. Urban T. A. Lazar N. A. Sweeney J. A. (2004). Maturation of cognitive processes from late childhood to adulthood. *Child Dev.* 75 1357–1372. 10.1111/j.1467-8624.2004.00745.x 15369519

[B53] Luna B. Wright C. (2016). “Adolescent brain development: Implications for the juvenile criminal justice system,” in *APA Handbooks in Psychology Series. APA Handbook of Psychology and Juvenile Justice*, eds Heilbrun K. DeMatteo D. Goldstein N. E. S. (Washington, DC: American Psychological Association), 91–116. 10.1037/14643-005

[B54] MacPherson L. Magidson J. F. Reynolds E. K. Kahler C. W. Lejuez C. W. (2010). Changes in sensation seeking and risk-taking propensity predict increases in alcohol use among early adolescents. *Alcohol. Clin. Exp. Res* 34 1400–1408. 10.1111/j.1530-0277.2010.01223.x 20491737 PMC3123723

[B55] McCrae R. R. Costa P. T. Ostendorf F. Angleitner A. Hřebíčková M. Avia M. D. (2000). Nature over nurture: temperament, personality, and life span development. *J. Pers. Soc. Psychol.* 78 173–186. 10.1037/0022-3514.78.1.173 10653513

[B56] Mitchell S. H. Schoel C. Stevens A. A. (2008). Mechanisms underlying heightened risk taking in adolescents as compared with adults. *Psychon. Bull. Rev.* 15 272–277. 10.3758/PBR.15.2.272 18488639 PMC2825152

[B57] Nook E. C. Sasse S. F. Lambert H. K. Mclaughlin K. A. Somerville L. H. (2017). Increasing verbal knowledge mediates development of multidimensional emotion representations. *Nat. Hum. Behav.* 1 881–889. 10.1038/s41562-017-0238-7 29399639 PMC5790154

[B58] Nucci M. Mapelli D. Mondini S. (2012). Cognitive Reserve Index questionnaire (CRIq): a new instrument for measuring cognitive reserve. *Aging Clin. Exp. Res.* 24 218–226. 10.3275/7800 21691143

[B59] Paus T. (2005). Mapping brain maturation and cognitive development during adolescence. *Trends Cogn. Sci.* 9 60–68. 10.1016/j.tics.2004.12.008 15668098

[B60] Raven J. C. Court J. H. Raven J. (1985). *A Manual for Raven’s Progressive Matrices and Vocabulary Scales.* London: H. K. Lewis.

[B61] Reyna V. F. Estrada S. M. DeMarinis J. A. Myers R. M. Stanisz J. M. Mills B. A. (2011). Neurobiological and memory models of risky decision making in adolescents versus young adults. *J. Exp. Psychol. Learn. Mem. Cogn.* 37 1125–1142. 10.1037/a0023943 21707215

[B62] Reyna V. F. Farley F. (2006). Risk and rationality in adolescent decision making. *Psychol. Sci. Public Interest* 7 1–44. 10.1111/j.1529-1006.2006.00026.x 26158695

[B63] Romer D. Reyna V. F. Satterthwaite T. D. (2017). Beyond stereotypes of adolescent risk taking: placing the adolescent brain in developmental context. *Dev. Cogn. Neurosci.* 27 19–34. 10.1016/j.dcn.2017.07.007 28777995 PMC5626621

[B64] Sherman L. Steinberg L. Chein J. (2017). Connecting brain responsivity and real-world risk taking: strengths and limitations of current methodological approaches. *Dev. Cogn. Neurosci.* 33 27–41. 10.1016/j.dcn.2017.05.007 28774477 PMC5745301

[B65] Shulman E. P. Harden K. P. Chein J. M. Steinberg L. (2015). Sex differences in the developmental trajectories of impulse control and sensation-seeking from early adolescence to early adulthood. *J. Youth Adolesc.* 44 1–17. 10.1007/s10964-014-0116-9 24682958

[B66] Shulman E. P. Smith A. R. Silva K. Icenogle G. Duell N. Chein J. (2016). The dual systems model: review, reappraisal, and reaffirmation. *Dev. Cogn. Neurosci.* 17 103–117. 10.1016/j.dcn.2015.12.010 26774291 PMC6990093

[B67] Smith A. R. Chein J. Steinberg L. (2013). Impact of socio-emotional context, brain development, and pubertal maturation on adolescent risk-taking. *Hormones Behav* 64 323–332. 10.1016/j.yhbeh.2013.03.006 23998675 PMC3761223

[B68] Sowell E. R. Trauner D. A. Gamst A. Jernigan T. L. (2002). Development of cortical and subcortical brain structures in childhood and adolescence: a structural MRI study. *Dev. Med. Child Neurol.* 44 4–16. 10.1111/j.1469-8749.2002.tb00253.x 11811649

[B69] Spear L. P. (2000). The adolescent brain and age-related behavioral manifestations. *Neurosci. Biobehav. Rev.* 24 417–463. 10.1016/S0149-7634(00)00014-2 10817843

[B70] Stadler C. Schmeck K. Janke W. (2004). *Inventar zur Erfassung von Impulsivität, Risikoverhalten und Empathie bei 9-bis 14-jährigen Kindern: IVE.* Oxford: Hogrefe.

[B71] Steinberg L. (2008). A social neuroscience perspective on adolescent risk-taking. *Dev. Rev.* 28 78–106. 10.1016/j.dr.2007.08.002 18509515 PMC2396566

[B72] Steinberg L. Albert D. Cauffman E. Banich M. Graham S. Woolard J. (2008). Age differences in sensation seeking and impulsivity as indexed by behavior and self-report: evidence for a dual systems model. *Dev. Psychol.* 44 1764–1778. 10.1037/a0012955 18999337

[B73] Urošević S. Collins P. Muetzel R. Lim K. Luciana M. (2012). Longitudinal changes in behavioral approach system sensitivity and brain structures involved in reward processing during adolescence. *Dev. Psychol.* 48 1488–1500. 10.1037/a0027502 22390662 PMC3370133

[B74] van den Bos W. Hertwig R. (2017). Adolescents display distinctive tolerance to ambiguity and to uncertainty during risky decision-making. *Sci. Rep.* 7:40962. 10.1038/srep40962 28098227 PMC5241878

[B75] Van Duijvenvoorde A. C. K. Jansen B. R. J. Bredman J. C. Huizenga H. M. (2012). Age-related changes in decision making: Comparing informed and noninformed situations. *Dev. Psychol.* 48 192–203. 10.1037/a0025601 21967563

[B76] Willoughby T. Good M. Adachi P. J. C. Hamza C. Tavernier R. (2013). Examining the link between adolescent brain development and risk taking from a social–developmental perspective. *Brain Cogn.* 83 315–323. 10.1016/j.bandc.2013.09.008 24128659

[B77] Zuckerman M. (2007). *Sensation Seeking and Risky Behavior.* Washington, DC: APA Books.

